# Using a Geosocial Networking App to Investigate New HIV Infections and Related Risk Factors Among Student and Nonstudent Men Who Have Sex With Men in Chengdu, China: Open Cohort Study

**DOI:** 10.2196/43493

**Published:** 2023-07-28

**Authors:** Zhen Dai, Guodong Mi, Fei Yu, Guodong Chen, Xiaodong Wang, Qinying He

**Affiliations:** 1 Department of Sexually Transmitted Disease AIDS Prevention and Control Chengdu Center for Disease Control and Prevention Chengdu China; 2 BlueCity Holdings Beijing China; 3 Department of Anthropology University of Copenhagen Copenhagen Denmark; 4 Chengdu Tongle Social Work Service Center Chengdu China

**Keywords:** geosocial networking app, GSN app, young men who have sex with men, MSM, HIV, incidence, risk factors, cohort study, smartphone, mobile phone

## Abstract

**Background:**

In China, condomless sex among men who have sex with men (MSM) is the primary route of HIV infection in young people. Chengdu is a hotspot for reported HIV cases among young people nationwide. Extensive use of geosocial networking (GSN) smartphone apps has dramatically changed the pattern of sexual behavior among young MSM (YMSM). However, data on HIV incidence and the risk behavior of YMSM using the GSN app are still obscure.

**Objective:**

This study aims to analyze and understand the HIV incidence and its risk factors among YMSM using GSN apps in Chengdu, China.

**Methods:**

An open cohort study was conducted among YMSM aged 18-24 years through a gay GSN smartphone app in Chengdu, China, from July 2018 to December 2020. Every participant completed a web-based questionnaire on sociodemographic characteristics, sexual behaviors, and other related statuses; made a reservation for a web-based HIV testing; and then voluntarily got tested at the designated testing site. At least one additional HIV test was taken via the app during the study period, and participants were evaluated at the end of the study or at the time of HIV seroconversion. By dividing the sum of the observed HIV seroconversions by the observed person-years, HIV incidence was calculated and compared between the student and nonstudent MSM. Univariate and multivariate (Cox proportional hazards regression) analyses were used to discuss the risk factors for new HIV infections.

**Results:**

In the study cohort, 24 seroconversions occurred among 625 YMSM who took at least two HIV tests through the app during the study period, contributing to 505 observed person-years. The HIV incidence rate per 100 person-years was 4.75 (95% CI 2.89-6.61) among all MSM, 3.60 (95% CI 1.27-5.93) among student MSM, and 5.88 (95% CI 2.97-8.79) among nonstudent MSM. In addition, the HIV incidence per 100 person-years was 11.11 (95% CI 4.49-17.73) among those who had resided in the area for 6 months or less and 7.14 (95% CI 1.52-12.77) among those with senior high school or less education. Two or more sexual partners (adjusted hazards ratio [HR] 3.63, 95% CI 1.08-12.23) in the preceding 6 months was a risk factor for new HIV infections. Consistent condom use for anal sex (adjusted HR 0.38, 95% CI 0.16-0.88) and insertive anal sex only (adjusted HR 0.10, 95% CI 0.01-0.75) in the preceding 6 months were protective factors for new HIV infections.

**Conclusions:**

The rate of new HIV infections among YMSM who actively used GSN smartphone apps was high, especially among migrant nonstudent MSM. Targeted interventions on GSN smartphone apps should be implemented to provide demand-adapted prevention and services to reduce the threat of HIV.

## Introduction

### Background

Globally, the population-wide morbidity and mortality of HIV infection has declined significantly; however, that the 15-24 years age group, defined as youth by the United Nations, still experience rising HIV infections, with 4000 new HIV infections per day in 2020, and 31% of these were among young people in this age group [1,2]. In some Asia-Pacific regions, the epidemic outbreaks are mostly among men who have sex with men (MSM), with more than a quarter of new infections occurring in the youth. Data from Indonesia, Pakistan, and the Philippines show the vulnerability of young MSM (YMSM: aged 18-24 years) to HIV transmission [1]. Understanding HIV incidence, particularly among subgroups of key populations, is essential for efficiently targeting strategies to prevent new HIV infections. However, previous studies in China have focused on HIV prevalence and related risk behavior among YMSM, with only a few reporting the HIV incidence among different sociodemographic groups of YMSM.

There are wide variations in HIV incidence according to the limited data from cohort studies. A cross-sectional study in China with data from 2012 to 2013 reported HIV incidences to be 11.8/100 person-years among all YMSM, 10 per 100 person-years among student YMSM, and 12.9/100 person-years among nonstudent YMSM [3,4]. Only 1 cohort observed an incidence of 6.7/100 person-years for YMSM in 8 cities of China in 2014 [5].

In 2019, the number of reported HIV-infected individuals and patients with AIDS among students and nonstudents aged 15-24 years in China was 3422 and 12,368, respectively. These figures were 4.3 and 1.4 times higher than those in 2010 [6,7]. Furthermore, the majority of reported cases in this group were aged 20-24 years, accounting for 76% to 84.3% of the infections [6]. Unprotected homosexual intercourse was the main route of infection for reported HIV cases in the youth, accounting for more than 80% of reported student cases [8] and 70%-80% of nonstudent cases in the same year [9,10]. Chengdu, the capital city of Sichuan province and the economic and cultural center of southwest China, has become a megacity with a resident population of over 20 million [11], of which the floating population accounts for 40.4% [12]. It is also a concentration of colleges and universities that accommodate nearly 1 million college students [13], and it served as a gathering place for about 120,000 YMSM in 2018 [14]. It has been a hot spot for reported HIV cases aged 15-24 years [6,7,15]. However, data are lacking on new HIV infections among YMSM with different characteristics in Chengdu.

Because of the dual status of in-school YMSM as a student and a gay man and the high mobility of off-school YMSM, it was very challenging to maintain a YMSM cohort. As a consequence, new strategies for recruiting and retaining YMSM are needed. With the rise of the mobile internet, the geosocial networking (GSN) app is widely used among the youth. Blued, a popular GSN smartphone app for YMSM, has about 3 million monthly active users aged 18-25 years [16,17]. The average number of monthly active users has exceeded 160,000 within 6 months in the immediate metropolitan area of Chengdu. On the one hand, this situation has made it possible to build an internet-based YMSM cohort that is more efficient and cost-effective than site recruitment [18]. On the other hand, it has also made it easier for YMSM to find sexual partners via the GSN smartphone app. A previous survey shows that 80% of YMSM aged 15-24 years in Chengdu have had male sexual partners in the past 6 months, with most of these encounters originating from mobile social networking platforms [19]. Previous studies have shown inconsistent results on whether MSM who use GSN smartphone apps have a higher risk of HIV infection. Some studies suggest that GSN app users take more recreational drugs and have more casual partners as well as condomless anal intercourse compared to nonusers [20-23], and that increases the risk of HIV infection [20,24,25]. It has also been suggested that MSM who use GSN apps are more aware of condom use [26], conduct HIV testing more frequently [27], and do not increase the risk of HIV infection [28,29]. The risk of HIV infection and risky sexual behavior among YMSM who use GSN smartphone apps is even less clear. Thus, reliable data on the HIV incidence and the changing sexual behaviors of GSN app-using YMSM are imperatively needed.

### Objectives

In this context, our study aims to address these gaps. More specifically, our study tries to (1) construct the first gay GSN app–using YMSM cohort in Chengdu, (2) generate knowledge of HIV incidence and its related risk factors among student and nonstudent MSM who use GSN smartphone apps, and (3) summarize the behavioral characteristics of YMSM who use GSN smartphone apps.

## Methods

### Study Design

Blued app users can voluntarily make an appointment for an HIV test on the app, which directly provides the location and time for rapid testing. The specific process has been reported in previous studies [18,30]. That makes YMSM who make an appointment for an HIV test through Blued an unparalleled natural cohort. Based on this, we constructed an open cohort of YMSM since June 2018 in Chengdu. Users were invited to participate in the cohort and asked to complete a self-administered web-based questionnaire before each testing appointment. The questionnaire included sociodemographic characteristics, sexual behaviors, recreational drug use, sexually transmitted infections (STIs) status, and HIV testing history. Fingertip blood was collected at the rapid testing site for HIV antibody test, and once positive, venous blood was taken and sent to the designated laboratory for a Western blot confirmatory test. In addition, whenever participants visited the GSN app for an HIV test, they could complete a follow-up, and new participants were enrolled when they visited the app for a test.

Since July 1, 2018, the first record of HIV testing, along with the web-based questionnaire on Blued, was taken as the baseline data; subsequent testing records and web-based questionnaires were considered to be follow-up observations. The evaluation was applied at the time of HIV seroconversion or the end of the study (December 31, 2020), whichever came first. Those who took at least two HIV tests through Blued during the study period had the time interval between their latest HIV test and the baseline test measured. Each positive HIV test result occurring during the follow-up in the cohort was counted as one seroconversion [18].

No incentives were offered for participation in our survey to ensure data quality. However, all participants received private messages, messages on the startup screen, advertisement banners, and invitations to live-streaming broadcasts through Blued. In these messages, YMSM were encouraged to test for HIV to reduce the loss to follow-up.

### Participant Eligibility Criteria

Young men who met the following criteria were recruited into our cohort: (1) born biologically male, (2) aged between 18 and 24 years, (3) had ever had sex with men, (4) resided in Chengdu with a Blued account registered in Chengdu, (5) completed the web-based survey and in-person HIV test during the study period, and (6) voluntarily participated in this study and signed the web-based informed consent. MSM were excluded if they (1) tested positive for HIV at baseline and (2) completed questionnaires within 30 seconds or had incomplete questionnaires.

### Outcomes and Measures

The primary outcome was the number of HIV seroconversion among different groups of YMSM. Two main categories of indicators were collected: sociodemographic characteristics and sexual behaviors in the preceding 6 months. Sociodemographic indicators included age (years), education level (senior high school or less and some college education or higher), occupation (student or nonstudent), and duration of residence in Chengdu (0-6 months or more than 6 months). Sexual behaviors included having anal sex intercourse (yes or no), number of sex partners (0 or 1, or 2 or more), having sex partners who had tested positive for HIV (yes, none, or not sure), frequency of condom use during anal sex (not every time or every time), role during anal sex (exclusively receptive, exclusively insertive, or versatile), commercial sex (yes or no), diagnosed with other STIs (yes or no), and recreational drug use (yes or no). We also collected participants’ HIV test history (yes or no).

### Statistical Analyses

We calculated the individual observation time as the interval between each participant’s baseline and their latest HIV test during the study period. By dividing the sum of the observed HIV seroconversions by the observed person-years, we calculated the HIV incidence and compared it between different subgroups. The Kaplan-Meier cumulative probability of HIV seroconversion between student MSM and nonstudent MSM was presented using survival curves. Participants’ sociodemographic characteristics, sexual behaviors, and HIV test history were summarized by descriptive statistics and compared between student and nonstudent MSM at the baseline survey. We converted quantitative variables, such as age and number of sexual partners during the last 6 months, into categorical variables. The HIV incidence was calculated by different groups of age, occupation, duration of residence in Chengdu, and education level. In addition, a proportional hazards regression model was developed for multivariate analysis using independent variables with *P*<.10 in the univariate analysis to identify the related risk factors. Covariates with *P*<.05 were contained in the final model. Moreover, in the model, age, occupation, sexual behaviors, and HIV test history were used as independent variables, while HIV seroconversions were used as dependent variables. Statistical analysis was performed using SAS software (version 9.4).

### Ethics Approval

The study protocol was reviewed and approved by the Chengdu Center for Disease Control and Prevention Ethics Committee (ethical review number of 2020003). Electronic individual informed consent was obtained before the web-based survey. For analysis, data on desensitization were exported from Blued, which did not contain personal information. The procedures in the study were performed in accordance with the study protocol and relevant regulations.

## Results

### The Cohort and HIV Seroconversion

**Figure 1:**
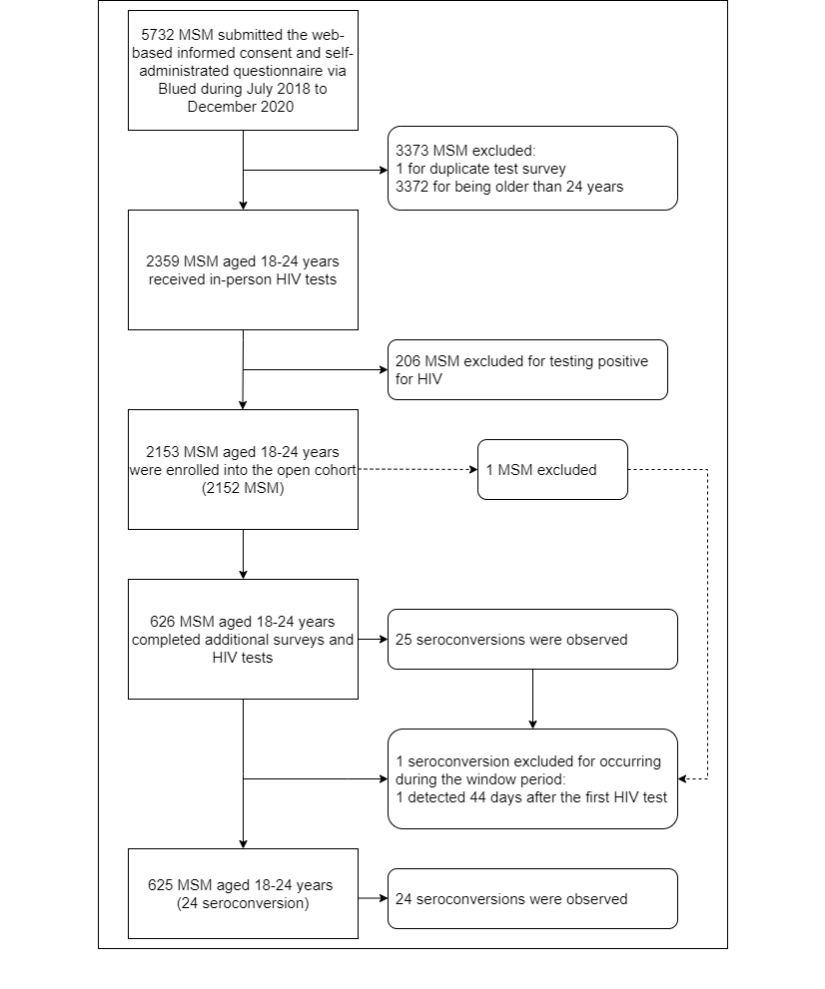
Flowchart of the study population selection for the geosocial networking app using men who have sex with men (MSM) in Chengdu, China.

A total of 2359 MSM aged between 18 and 24 years completed the web-based informed consent and questionnaire and received in-person HIV testing at baseline. After excluding 206 MSM who screened positive, we included 2153 participants aged 18-24 years who tested negative in the cohort at baseline. Following the exclusion of 1 seroconversion that occurred within the window period (≤45 days), our cohort identified 24 HIV seroconversions among 625 YMSM who reported 2 or more episodes of HIV testing during the study period, comprising 9 seroconversions among 313 students and 15 seroconversions among 312 nonstudents. Figure 1 illustrates the population selection for our study.

In total, 1527 YMSM who tested negative for HIV did not take 2 or more tests through the GSN app during the study period. We did sensitive analyses on sociodemographic characteristics, sexual behaviors, STIs, recreational drug use in the last 6 months, and HIV test history between the groups of YMSM who tested twice or more and those who only tested once. We found that the 2 groups were significantly different in some key factors; the rate of anal sex with men were higher for the former group (547/625, 87.5%) than the latter (1224/1527, 80.2%), as well as having multiple sexual partners (361/625, 57.8% vs 716/1527, 46.9%). Moreover, the proportion of ever tested for HIV was also higher in the former group (463/625, 74.1% vs 936/1527, 61.3%; Table S1 in Multimedia Appendix 1).

**Figure 2:**
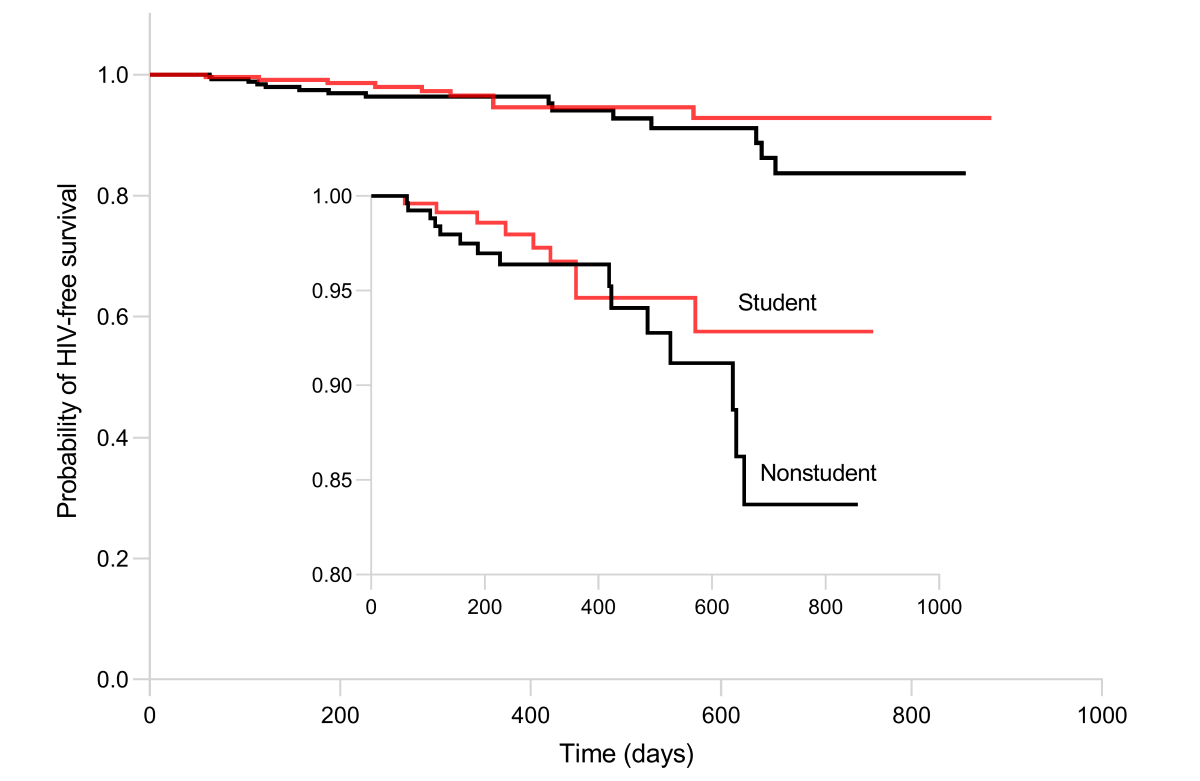
HIV incidence among student and nonstudent men who have sex with men in Chengdu and the probability of HIV-free survival.

The follow-up interval ranged from 15 to 884 days, with a median of 244 (IQR 95-437) days. Furthermore, the total person-years observed was 250 for student and 255 for nonstudent MSM. Figure 2 reveals the Kaplan-Meier cumulative probability of HIV-free survival. Although there was no statistically significant difference between student and nonstudent MSM, the probability of HIV-free survival in the nonstudent group decreased rapidly near the end of the study.

### Demographic Characteristics, Sexual Behaviors, and HIV Test History of the Student and Nonstudent MSM Participants

The median age of cohort participants was 22 (IQR 20-23) years, with a median age of 21 (IQR 19-22) years for students and 22 (IQR 21-23) years for nonstudents. Compared with nonstudents, students showed the following distinct characteristics: age 18-21 years (201/313, 64.2% vs 102/312, 32.7%), lived in Chengdu for more than 6 months (249/313, 79.6% vs 234/312, 75%), and some college education level or higher (286/313, 91.4% vs 227/312, 72.8%). In terms of sexual behavior in the past 6 months, the performance of students and nonstudents was as follows: receptive anal sex only (112/264, 42.4% vs 86/283, 30.4%), commercial sex (5/313, 1.6% vs 18/312, 5.8%), and recreational drug use (48/313, 15.3% vs 55/312, 17.6%). The proportion of students with a history of HIV testing was lower than that of nonstudents (204/313, 65.2% vs 259/312, 83%; Table 1).

**Table 1:** Sociodemographic characteristics, sexual behaviors, sexually transmitted infections (STIs), recreational drug use in the last 6 months and HIV test history of the student and nonstudent men who have sex with men.

Characteristics		Student	Nonstudent	*χ* ^2^ *(df)*		*P* value	
**Age (years), n (%)**					62.5 (2)		<.001
	18-19	82 (26.2)	38 (12.2)				
	20-21	119 (38)	64 (20.5)				
	22-24	112 (35.8)	210 (67.3)				
Age (years), median (IQR)		21 (19-22)	22 (21-23)				
**Duration of residence in Chengdu** **, n (%)**					1.8 (1)		.17
	0-6 months	64 (20.4)	78 (25)				
	More than 6 months	249 (79.6)	234 (75)				
**Education level** **, n (%)**					36.8 (1)		<.001
	Senior high school or less	27 (8.6)	85 (27.2)				
	Some college education or higher	286 (91.4)	227 (72.8)				
**Anal sex with men** **in the last 6 months** **, n (%)**					5.8 (1)		.02
	Yes	264 (84.3)	283 (90.7)				
	No	49 (15.7)	29 (9.3)				
**Using condom for anal sex** **in the last 6 months** **, n (%)**					0.03 (1)		.85
	Consistent	150 (56.8)	163 (57.6)				
	Inconsistent	114 (43.2)	120 (42.4)				
**Sex role for anal sex** **in the last 6 months** **, n (%)**					10.9 (2)		.004
	Exclusively receptive	112 (42.4)	86 (30.4)				
	Exclusively insertive	66 (25)	102 (36)				
	Versatile	86 (32.6)	95 (33.6)				
**Number of sexual partners** **in the last 6 months** **, n (%)**					2.5 (1)		.11
	0-1	142 (45.4)	122 (39.1)				
	≥2	171 (54.6)	190 (60.9)				
**HIV-positive partners** **in the last 6 months** **, n (%)**					2.9 (2)		.24
	None	115 (36.7)	130 (41.7)				
	Yes	16 (5.1)	21 (6.7)				
	Not sure	182 (58.1)	161 (51.6)				
**Commercial sex** **in the last 6 months** **, n (%)**					7.7 (1)		.006
	Yes	5 (1.6)	18 (5.8)				
	No	308 (98.4)	294 (94.2)				
**Diagnosed with a STI** **in the last 6 months** **, n (%)**					1.8 (1)		.18
	Yes	11 (3.5)	18 (5.8)				
	No	302 (96.5)	294 (94.2)				
**Recreational drug use** **in the last 6 months** **, n (%)**					0.6 (1)		.44
	Yes	48 (15.3)	55 (17.6)				
	No	265 (84.7)	257 (82.4)				
**HIV test history** **, n (%)**					25.9 (1)		<.001
	Yes	204 (65.2)	259 (83)				
	No	109 (34.8)	53 (17)				

### The HIV Incidence

Table 2 shows the HIV incidence of different sociodemgrahic subgroups of cohort participants. The HIV incidence rate per 100 person-years was 4.75 (95% CI 2.89-6.61) among all MSM, 3.60 (95% CI 1.27-5.93) among student MSM, and 5.88 (95% CI 2.97-8.79) among nonstudent MSM. Compared with YMSM who lived in Chengdu for more than 6 months, the HIV incidence rate of those who lived in Chengdu for 6 months or less was higher (11.11, 95% CI 4.49-17.73 vs 3.37, 95% CI 1.63-5.12; *P*=.007). The HIV incidence rate of those with senior high school or less education level looked higher compared to those with some college education or higher, with no statistical significance (7.14, 95% CI 1.52-12.77 vs 4.28, 95% CI 2.34-6.22; *P*=.29).

**Table 2:** The HIV incidence of different sociodemgrahic characteristics of the study cohort.

Characteristics			MSM^a^, n (%)		Observed person-years, n		Seroconversion, n		HIV incidence rate per 100 person-years (95% CI)
**Age (years)**									
	18-19	120 (19.2)		98		5		5.10 (0.67-9.53)	
	20-21	183 (29.3)		140		6		4.29 (0.89-7.68)	
	22-24	322 (51.5)		267		13		4.87 (2.27-7.47)	
**Occupation**									
	Student	313 (50.1)		250		9		3.60 (1.27-5.93)	
	Nonstudent	312 (49.9)		255		15		5.88 (2.97-8.79)	
**Duration of residence in Chengdu**									
	0-6 months	142 (22.7)		90		10		11.11 (4.49-17.73)	
	More than 6 months	483 (77.3)		415		14		3.37 (1.63-5.12)	
**Education level**									
	Senior high school or less	112 (17.9)		84		6		7.14 (1.52-12.77)	
	Some college education or higher	513 (82.1)		421		18		4.28 (2.34-6.22)	
	All	625 (100)		505		24		4.75 (2.89-6.61)	

^a^MSM: men who have sex with men.

### Analyses of Risk Factors Associated With HIV Seroconversion

In univariate analysis, sexual behaviors in the past 6 months, including a higher number of sexual partners, inconsistent condom use during anal sex, and exclusively receptive anal sex, were associated with incident HIV (*P*<.05; Table 3). In multivariable analysis, having 2 or more sexual partners (adjusted hazards ratio [HR] 3.63, 95% CI 1.08-12.23) in the preceding 6 months was considered a risk factor for new HIV infections, compared with a single sexual partner. Consistent condom use for anal sex (adjusted HR 0.38, 95% CI 0.16-0.88) and insertive anal sex only (adjusted HR 0.10, 95% CI 0.01-0.75) in the preceding 6 months were identified as protective factors against new HIV infections. Having partners who had tested positive for HIV, engaging in commercial sex, being diagnosed with STIs, recreational drugs use, and having an HIV test history were not associated with new HIV infections (*P*>.05).

**Table 3:** Factors associated with HIV seroconversion were identified using a proportional hazards regression model.

Factor			Bivariate model					Multivariable model		
			Hazards ratio (95% CI)		*P* value		Adjusted hazards ratio (95% CI)			*P* value
**Age (years)**										N/A^a^
	18-19	Reference		N/A		N/A				
	20-21	0.85 (0.26-2.79)		.79		N/A				
	22-24	0.96 (0.34-2.69)		.93		N/A				
**Occupation**										N/A
	Student	Reference		N/A		N/A				
	Nonstudent	1.62 (0.71-3.70)		.25		N/A				
**Anal sex with men** **in the last 6 months**										N/A
	Yes	Reference		N/A		N/A				
	No	0.33 (0.05-2.48)		.28		N/A				
**Using condom for anal sex** **in the last 6 months**										
	Inconsistent	Reference		N/A		Reference			N/A	
	Consistent	0.41 (0.18-0.94)		.04		0.38 (0.16-0.88)			.02	
**Sex role for anal sex** **in the last 6 months**										
	Exclusively receptive	Reference		N/A		Reference			N/A	
	Exclusively insertive	0.10 (0.01-0.77)		.03		0.10 (0.01-0.75)			.03	
	Versatile	1.02 (0.44-2.35)		.97		1.02 (0.44-2.35)			.97	
**Number of sexual partners** **in the last 6 months**										
	0-1	Reference		N/A		Reference			N/A	
	≥2	3.64 (1.08-12.24)		.04		3.63 (1.08-12.23)			.04	
**HIV-positive partners** **in the last 6 months**										N/A
	None	Reference		N/A		N/A				
	Yes	0.79 (0.10-6.18)		.82		N/A				
	Not sure	0.89 (0.39-2.02)		.77		N/A				
**Commercial sex** **in the last 6 months**										N/A
	Yes	Reference		N/A		N/A				
	No	0.34 (0.08-1.44)		.14		N/A				
**Diagnosed with a sexually transmitted infection** **in the last 6 months**										N/A
	Yes	Reference		N/A		N/A				
	No	0.49 (0.12-2.09)		.34		N/A				
**Recreational drug use** **in the last 6 months**										N/A
	Yes	Reference		N/A		N/A				
	No	0.49 (0.20-1.19)		.12		N/A				
**HIV test history**										N/A
	Yes	Reference		N/A		N/A				
	No	1.16 (0.46-2.94)		.75		N/A				

^a^N/A: not applicable.

## Discussion

### Advantages of Maintaining a Cohort Through a GSN App

This innovative method of using GSN apps to maintain a cohort offers advantages in reaching high-risk YMSM and overcoming barriers related to stigma and discrimination associated with in-person cohort studies. In particular, student MSM, who have a dual identity as students and gay men, are difficult to contact in person. Moreover, an electronic questionnaire loaded on the app may enhance the data quality of research related to sexual and highly stigmatized behaviors among YMSM [18]. Surveys on sensitive issues, such as sexuality, can be conducted web based to avoid embarrassment and discrimination in in-person, face-to-face settings. Moreover, this is based on studies that have reported increased self-disclosure of sensitive information with decreased personal interactions with an interviewer [31]. We conducted our research using a popular GSN app among Chinese YMSM for socializing, through which they could easily and quickly access to HIV-related counseling and testing services when needed.

### Significance of the Study Results

Studies indicate that GSN apps increase HIV risk in MSM by facilitating sexually risky behaviors [20-25]. In our study, the HIV incidence of app-using YMSM is higher, compared to the data (3.09/100 person-years) of MSM from the most significant local nongovernmental organization serving in Chengdu for voluntary counseling and testing services through web-based and in-person routes in 2018 [32]. That may be because our participants are young active users of GSN apps, where they could easily and quickly find potential sexual partners. However, making any direct comparison of the incidence between our cohort and others is not recommended, considering the differences in the establishment, age groups, and follow-up methods. Reported HIV incidence may vary because of different periods and regions, progressive intervention efforts, changing patterns of HIV risk behaviors among subpopulations of MSM, as well as differences in recruitment and follow-up methods. We found a high rate of new HIV infections among newly adult, low-education level, and out-of-school YMSM, especially among young migrants who had recently settled in Chengdu. Out-of-school YMSM who have just reached adulthood are vulnerable to HIV infection. Due to their special stage of physical and mental development, lack of social support and school education, limited economic income, and weak health awareness, coupled with discrimination and stigma, they do not know enough about AIDS and sexually transmitted diseases. They have poor compliance with interventions and treatments [33]. Studies have found that mobility experiences increase the occurrence of risky behaviors, such as substance abuse, commercial sex, condomless sex, and multiple sexual partners, among out-of-school YMSM. Furthermore, the mobility across provinces and cities also affects access to health services and makes it more difficult to provide comprehensive HIV prevention, treatment, and intervention services [34].

Most of the factors associated with HIV seroconversion in the study were consistent with other studies using traditional methods. In addition to using condoms during sex, having multiple sexual partners is an independent risk factor for acquiring HIV [35,36]. Multiple sexual partners are also widely recognized as a predictor of STI acquisition [37]. In China [22] and other countries [21,24], GSN app users reported having more sexual partners than nonusers. Users of the GSN smartphone app could easily find potential sexual partners nearby, leading to more casual sex and multiple sexual partners, facilitating condomless anal sex [20-23]. We found that inconsistent condom use for anal sex and multiple sexual partners increased the probability of HIV incidence, which was consistent with another study conducted among MSM in Chengdu [32]. The risk of HIV infection for MSM exclusively practicing insertive anal sex was reduced in our study, in agreement with other studies [38,39]. It was found that MSM who only engaged in insertive anal sex had a lower risk of contracting HIV in the United States (HR 0.55, 95% CI 0.36-0.84) [40].

### HIV Intervention Strategies for App-Using YMSM

The findings indicate that YMSM, both in and out of school, face multiple challenges related to sexual health, including the risk of STIs from unsafe sex, which threatens their healthy development. Therefore, it is imperative to address the issue of reducing the risk of HIV infection in YMSM. Prevention or treatment service programs for this population need to be adapted to the needs of current and future developments, combining prevention education with comprehensive sexual and reproductive health education [41]. The internet and GSN smartphone app are important channels for disseminating HIV information and knowledge and promoting counseling and testing services to student and nonstudent MSM. Student MSM can implement precise intervention and peer education through GSN app positioning. It is challenging to educate nonstudent MSM because they lack access to important knowledge in schools. It is essential to make full use of the internet and social networking platforms to improve the accessibility of health education and to carry out intervention and treatment services that meet the needs and adapt to the characteristics of this population [42].

### Study Strengths

A major strength of this study is its innovative use of the GSN app in constructing and maintaining a cohort of student and nonstudent MSM in China. We were able to construct an open cohort to estimate HIV incidence among YMSM who use the GSN app, especially among those with different sociodemographic traits. As long as participants accessed the GSN app for an HIV test, they could complete the follow-up, making our cohort more natural and representative. In addition, we are still enrolling and following up with our cohort, so more data will be collected to improve the robustness of future studies.

### Limitations

Several limitations exist in our study. First, our study population consisted of YMSM seeking partners and health services through GSN apps. Participants who had repeated HIV test records had a higher rate of risky sexual behaviors. Therefore, the findings of our study cannot be generalized to the entire YMSM in Chengdu, such as those who do not use the apps. Second, we did not follow participants and did not them to do the test at a fixed time. Instead, all of them chose the test according to their situation and voluntariness. There was a natural loss of follow-up, and they constituted a relatively less active population. Therefore, our estimate of HIV incidence might be slightly overestimated. Third, although we found different HIV incidences among different YMSM subgroups, the factors influencing new HIV infections were not explored due to the reduced sample size and low statistical power after grouping. Thus, a larger prospective cohort is needed to further examine the risk factors of new HIV infections in GSN app–using YMSM with different sociodemographic characteristics.

### Conclusions

YMSM who use a GSN smartphone app in Chengdu had a high HIV incidence, which may be influenced by their HIV-related high-risk behavior, including multiple sexual partners and inconsistent condom use. Especially migrant nonstudent MSM had even higher HIV incidence compared to other subgroups. Thus, to mitigate the HIV epidemic among YMSM, government departments, such as health and education, should collaborate with GSN app operators to develop integrated web-based and in-person HIV intervention strategies for users of these platforms.

## Appendix

Multimedia Appendix 1 Table presenting sociodemographic characteristics, sexual behaviors, sexually transmitted infections, and recreational drug use in the last 6 months and HIV test history of the study cohort at baseline.

## Abbreviations

GSN: geosocial networking
HR: hazards ratio
MSM: men who have sex with men
STI: sexually transmitted infection
YMSM: young men who have sex with men

## Data Availability

The data sets used during this study are available from the corresponding author on reasonable request.
