# Supplementary material for: Using a Geosocial Networking App to Investigate New HIV Infections and Related Risk Factors Among Student and Nonstudent Men Who Have Sex With Men in Chengdu, China: Open Cohort Study
Source: J Med Internet Res. 2023 Jul 28;25:e43493. doi: 10.2196/43493 (PMC10422168; doi:10.2196/43493)
Supplement: Multimedia Appendix 1 [file jmir_v25i1e43493_app1.docx]

# Supplementary materials

**Table S1.** Sociodemographic characteristics, sexual behaviors, STIs, recreational drug use in the last 6 months and HIV test history of the cohort at baseline

| Characteristics | | Group of YMSM completed additional test | Group of YMSM rest | *χ^2^ (df)* | *P value* |
| --- | --- | --- | --- | --- | --- |
| **Age (years), n (%)** | | | | 2.8 (2) | .25 |
|  | 18-19 | 120 (19.2) | 338 (22.1) |  |  |
|  | 20-21 | 183 (29.3) | 452 (29.6) |  |  |
|  | 22-24 | 322 (51.5) | 737 (48.3) |  |  |
| **Occupation, n (%)** | | | | 0.2 (1) | .66 |
|  | Student | 313 (50.1) | 749 (49.1) |  |  |
|  | Nonstudent | 312 (49.9) | 778 (50.9) |  |  |
| **Duration of residence in Chengdu, n (%)** | | | | 6.1 (1) | .01 |
|  | 0-6 months | 142 (22.7) | 426 (27.9) |  |  |
|  | More than 7 months | 483 (77.3) | 1101 (72.1) |  |  |
| **Education level, n (%)** | | | | 2.3 (1) | .13 |
|  | Senior high school or less | 112 (17.9) | 318 (20.8) |  |  |
|  | Some college or higher | 513 (82.1) | 1209 (79.2) |  |  |
| **Anal sex with men in the last 6 months, n (%)** | | | | 16.5 (1) | <.001 |
|  | Yes | 547 (87.5) | 1224 (80.2) |  |  |
|  | No | 78 (12.5) | 303 (19.8) |  |  |
| **Using condom for anal sex in the last 6 months, n (%)** | | | | 0.06 (1) | .81 |
|  | Consistent | 313 (57.2) | 693 (56.6) |  |  |
|  | Inconsistent | 234 (42.8) | 531 (43.4) |  |  |
| **Sex role for anal sex in the last 6 months, n (%)** | | | | 5.5 (2) | .06 |
|  | Exclusively receptive | 198 (36.2) | 461 (37.7) |  |  |
|  | Exclusively insertive | 181 (33.1) | 451 (36.8) |  |  |
|  | Versatile | 168 (30.7) | 312 (25.5) |  |  |
| **Number of sexual partners in the last 6 months, n (%)** | | | | 21.0 (1) | <.001 |
|  | 0-1 | 264 (42.2) | 811 (53.1) |  |  |
|  | ≥2 | 361 (57.8) | 716 (46.9) |  |  |
| **HIV-positive partners in the last 6 months, n (%)** | | | | 12.2 (2) | .002 |
|  | None | 245 (39.2) | 669 (43.8) |  |  |
|  | Yes | 37 (5.9) | 46 (3.0) |  |  |
|  | Not sure | 343 (54.9) | 812 (53.2) |  |  |
| **Commercial sex in the last 6 months, n (%)** | | | | 4.1 (1) | .04 |
|  | Yes | 23 (3.7) | 89 (5.8) |  |  |
|  | No | 602 (96.3) | 1438 (94.2) |  |  |
| **Diagnosed with a sexually transmitted infection in the last 6 months, n (%)** | | | | 1.1 (1) | .29 |
|  | Yes | 29 (4.6) | 56 (3.7) |  |  |
|  | No | 596 (95.4) | 1471 (96.3) |  |  |
| **Recreational drug use in the last 6 months, n (%)** | | | | 1.3 (1) | .25 |
|  | Yes | 103 (16.5) | 222 (14.5) |  |  |
|  | No | 522 (83.5) | 1305 (85.5) |  |  |
| **HIV test history, n (%)** | | | | 31.9 (1) | <.001 |
|  | Yes | 463 (74.1) | 936 (61.3) |  |  |
|  | No | 162 (25.9) | 591 (38.7) |  |  |
